# Alternative Splicing in Plant Development and Abiotic Stress Responses: A Multifunctional Regulatory Mechanism

**DOI:** 10.3390/ijms27125512

**Published:** 2026-06-18

**Authors:** Hye-Yeon Seok, Sun-Young Lee, Dahyun Kim, Yong-Hwan Moon

**Affiliations:** 1 Institute of Systems Biology, Pusan National University, Busan 46241, Republic of Korea; seokhyeon@pusan.ac.kr (H.-Y.S.); symoonlee@pusan.ac.kr (S.-Y.L.); 2 Department of Integrated Biological Science, Pusan National University, Busan 46241, Republic of Korea; surreal_rego34@pusan.ac.kr; 3 Department of Molecular Biology, Pusan National University, Busan 46241, Republic of Korea

**Keywords:** alternative splicing, abiotic stress, developmental regulation, plant, splice isoform

## Abstract

Alternative splicing (AS) is a major post-transcriptional regulatory mechanism that greatly expands transcriptomic and proteomic diversity in plants. Recent studies have demonstrated that AS dynamically regulates gene expression during plant development and under diverse environmental conditions through isoform-specific modulation of transcript stability, translation efficiency, protein localization, and signaling pathways. In this review, we summarize recent advances in understanding the roles of AS in plant development and abiotic stress responses. Mechanistically, splice site selection is regulated through coordinated interactions among *cis*-regulatory elements, RNA-binding proteins, RNA secondary structures, transcriptional kinetics, chromatin organization, and spliceosomal dynamics. AS plays critical roles in various developmental processes, including seed germination, vegetative growth, flowering transition, and senescence, while also contributing to plant adaptation to abiotic stresses such as osmotic, temperature, and oxidative stresses. Particular emphasis is placed on the diverse regulatory outcomes of AS, including isoform-specific protein functions, AS-coupled nonsense-mediated decay, transcript stability control, and context-dependent isoform switching. We further discuss the varying levels of experimental evidence supporting reported AS events, ranging from transcriptome-wide observations to genetically and biochemically validated isoform functions. Moreover, recent advances in long-read sequencing, single-cell transcriptomics, proteogenomics, and genome-engineering technologies are accelerating the functional characterization of splice isoforms and uncovering the complexity of AS-mediated regulatory networks. Collectively, these advances highlight AS as a central mechanism coordinating plant developmental plasticity and environmental adaptation.

## 1. Introduction

Plants possess a high proportion of intron-containing genes, with nearly 90% of protein-coding genes interrupted by introns, making precursor messenger RNA (pre-mRNA) splicing an essential step in gene expression [1]. Alternative splicing (AS), which generates multiple mRNA isoforms from a single precursor transcript through differential splice site selection, is a major mechanism underlying the complexity of the eukaryotic transcriptome. Genome-wide transcriptome analyses have revealed that AS is highly prevalent in plants and affects more than 60% of intron-containing genes [2]. This widespread occurrence highlights its importance as a regulatory process that operates alongside transcriptional control. By producing distinct transcript isoforms, AS can alter protein structure and function, influence subcellular localization, and control transcript stability, thereby shaping gene expression outcomes [1]. In addition, the generation of transcripts containing premature termination codons can lead to their selective degradation, thereby linking AS to RNA quality-control pathways [2].

AS is dynamically regulated across developmental stages and in response to environmental cues. It plays important roles in diverse developmental processes, including organ formation, flowering time regulation, and circadian rhythm control, while also contributing to plant adaptation to abiotic stresses such as temperature fluctuations, drought, and salinity. These features suggest that AS enables the flexible modulation of gene expression programs under changing environmental conditions [1,3].

Despite significant advances in the transcriptome-wide identification of AS events, the functional relevance of many splice variants and the regulatory mechanisms governing AS remain incompletely understood. In this review, we summarize current knowledge of the molecular basis of AS and discuss its roles in plant development and abiotic stress responses, with a particular emphasis on isoform-level regulation and emerging regulatory principles. Unlike previous reviews that primarily focused on either developmental regulation or stress-responsive AS, we integrate these biological contexts to highlight the common regulatory mechanisms that underlie plant developmental plasticity and environmental adaptation. We also distinguish experimentally validated AS events from transcriptome-level observations and discuss how different levels of evidence contribute to our current understanding of AS function. By emphasizing both the mechanistic diversity and the varying degrees of functional validation across reported AS events, this review aims to provide a more critical perspective on the biological significance of AS in plants.

## 2. Molecular Basis of Alternative Splicing (AS) in Plants

### 2.1. Core Mechanisms of Precursor Messenger RNA Splicing

Pre-mRNA splicing is an essential post-transcriptional process that ensures the accurate removal of introns and ligation of exons to generate mature transcripts. In eukaryotes, this process is mediated by the spliceosome, a highly dynamic ribonucleoprotein complex composed of small nuclear RNAs and numerous associated proteins that recognize conserved sequence elements within pre-mRNAs, including the 5′ splice site, 3′ splice site, branch point sequence, and polypyrimidine tract [3,4].

The major spliceosome, which catalyzes the removal of U2-type introns, is composed of five small nuclear ribonucleoprotein particles (snRNPs): U1, U2, U4/U6, and U5. Spliceosome assembly proceeds through a highly coordinated and stepwise process [3,4]. Early spliceosome formation begins with recognition of the 5′ splice site by U1 snRNP and recruitment of U2 auxiliary factors (U2AFs) to the 3′ splice site region. Subsequent binding of U2 snRNP to the branch point sequence stabilizes splice site recognition and promotes assembly of the pre-catalytic spliceosomal complex. Dynamic rearrangements involving U4/U6 and U5 snRNPs subsequently generate the catalytically active spliceosome that mediates intron excision and exon ligation through coordinated RNA-RNA and RNA-protein interactions [3,4]. These transitions require extensive remodeling driven by ATP-dependent RNA helicases and spliceosome-associated accessory proteins [4].

RNA helicases play particularly important roles during spliceosome assembly and catalytic activation. DEAD-box and DEAH-box RNA helicases facilitate conformational rearrangements of RNA substrates and ribonucleoprotein complexes throughout the splicing cycle [5]. In *Arabidopsis thaliana* (*Arabidopsis*), multiple RNA helicases participate in pre-mRNA splicing, miRNA biogenesis, RNA surveillance, and stress-responsive RNA metabolism, highlighting the close integration of RNA structural remodeling with spliceosomal activity [5].

Liquid-liquid phase separation (LLPS) has recently emerged as a potential mechanism linking environmental signaling to post-transcriptional regulation. Many splicing factors and RNA helicases contain intrinsically disordered regions that promote the formation of dynamic nuclear condensates, including nuclear speckles. These condensates are thought to serve as local hubs that concentrate spliceosomal components and regulatory proteins, thereby facilitating efficient spliceosome assembly and splice site recognition. Under environmental stresses such as heat, drought, or oxidative stress, dynamic reorganization of these condensates may promote the selective recruitment or release of splicing regulators, thereby contributing to stress-responsive AS remodeling. Although the molecular mechanisms remain under active investigation, LLPS is increasingly recognized as an emerging regulatory framework that integrates environmental cues with RNA processing and spliceosome function in plants [6,7,8].

### 2.2. Types and Functional Consequences of AS

AS arises from the differential selection of splice sites within a single precursor transcript, generating multiple transcript isoforms from a single gene. Major forms of AS include intron retention, exon skipping, alternative 5′ splice site selection, alternative 3′ splice site selection, mutually exclusive exons, and alternative first- or last-exon usage. Among these AS types, intron retention is particularly prevalent in plants and represents a defining feature distinguishing plant AS landscapes from those of metazoans. Genome-wide transcriptomic analyses revealed that intron retention accounts for more than 60% of AS events in many plant species, including *Arabidopsis*, *Oryza sativa* (rice), and *Zea mays* (maize) [9].

Historically, retained introns were often considered splicing noise or nonfunctional intermediates. However, recent studies indicate that intron retention represents a highly heterogeneous and functionally important regulatory mechanism. In some cases, retained introns introduce premature termination codons, thereby targeting transcripts for nonsense-mediated decay (NMD) and regulating transcript abundance [3]. In other cases, intron-retaining transcripts remain stable. They are retained within the nucleus as “detained introns”, functioning as regulatory reservoirs that can undergo delayed splicing in response to developmental or environmental signals [10,11,12]. Stress-responsive intron-retaining transcripts may therefore provide a mechanism for rapid transcript activation without requiring de novo transcription. Recent proteogenomic analyses further revealed that a subset of intron-retaining transcripts can be translated, indicating that intron retention contributes to proteome diversification as well as transcript-level regulation [12].

Different forms of AS generate substantial functional diversity at both transcript and protein levels. Alternative exon usage and splice site selection can alter coding sequences, resulting in proteins with modified domain composition, catalytic activity, interaction specificity, or subcellular localization [13]. In plants, AS frequently modifies protein–protein interaction domains, DNA-binding regions, phosphorylation sites, or localization signals, thereby reshaping protein function without requiring transcriptional changes [13]. AS also influences untranslated regions (UTRs), thereby affecting mRNA stability, translational efficiency, RNA localization, and microRNA-mediated regulation. Changes in 5′ UTRs may alter translational initiation efficiency, whereas alternative 3′ UTRs can influence transcript stability and RNA-protein interactions. Through these mechanisms, AS contributes extensively to post-transcriptional regulation of gene expression [14].

The functional significance of AS at the proteome level has long been debated because many alternatively spliced transcripts were initially detected only at the RNA level. Recent large-scale proteogenomic studies in *Arabidopsis* identified thousands of isoform-specific peptides corresponding to alternatively spliced transcripts, providing direct evidence that many AS isoforms are translated [14]. However, despite increasing evidence for translation of alternatively spliced transcripts, the biological relevance of many isoforms remains incompletely understood. Numerous splice variants may exhibit condition-specific, low-abundance, or transient expression patterns, making functional validation particularly challenging. Therefore, a major goal of current AS research is to distinguish functional isoforms from transcriptional or splicing noise and to determine the physiological significance of individual AS events.

Collectively, these findings establish AS as a versatile regulatory mechanism that shapes transcriptome plasticity, proteome complexity, and gene-expression dynamics through the coordinated regulation of transcript processing, translation, and protein function. Importantly, the rapid expansion of transcriptome-wide AS datasets has substantially outpaced the functional characterization of individual splicing events. Consequently, the levels of evidence supporting reported AS functions vary considerably across studies. While some AS events have been validated through genetic, biochemical, proteomic, or phenotypic analyses that demonstrate isoform-specific biological functions, many others remain supported primarily by transcriptomic associations under specific developmental or environmental conditions (Table 1 and Table 2). Therefore, distinguishing experimentally validated AS functions from transcriptome-level observations is essential for accurately assessing the biological significance of AS. This distinction should be considered when interpreting the developmental and stress-responsive AS events discussed throughout this review.

### 2.3. Selection of Splice Site in AS

#### 2.3.1. *Cis*- and *Trans*-Regulatory Determinants in Splice Site Selection

**Figure 1:**
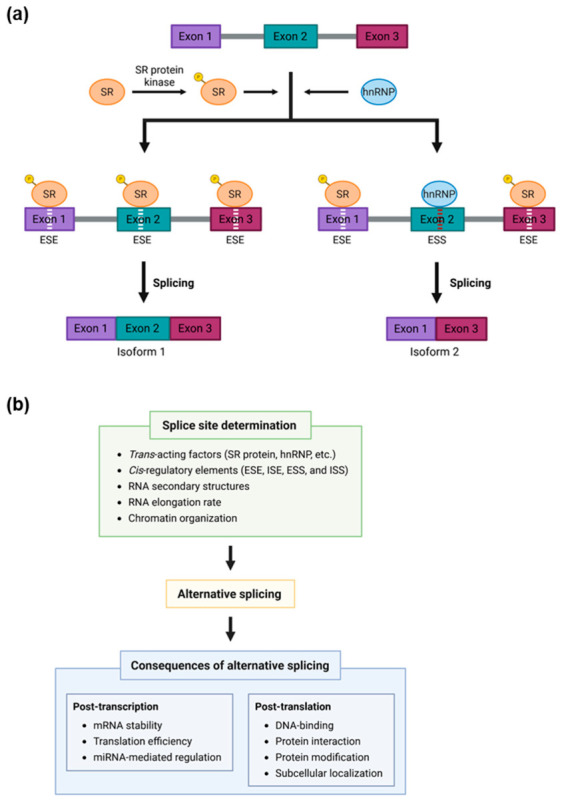
Regulatory mechanisms and functional consequences of AS in plants. (**a**) Representative mechanisms of splice site selection mediated by *cis*-regulatory elements and *trans*-acting splicing factors. Exonic splicing enhancers (ESEs) promote exon recognition by recruiting phosphorylated serine/arginine-rich (SR) proteins, resulting in exon inclusion. In contrast, binding of heterogeneous nuclear ribonucleoproteins (hnRNPs) to exonic splicing silencers (ESSs) suppresses splice site recognition and promotes exon skipping. These coordinated interactions between *cis*-regulatory elements and RNA-binding proteins (RBPs) determine splice site selection and generate distinct transcript isoforms during AS. Boxes and gray lines indicate exons and introns, respectively. (**b**) Schematic overview of the regulatory layers governing splice site determination and AS outcomes. Splice site selection is regulated through coordinated interactions among *cis*-regulatory elements, *trans*-acting factors, RNA secondary structures, chromatin organization, and RNA Polymerase II elongation kinetics. These regulatory mechanisms collectively shape AS patterns and contribute to diverse post-transcriptional and post-translational outcomes. Together, these multilayered regulatory processes enable AS to dynamically modulate gene expression during plant development and abiotic stress responses. Created in BioRender. Seok, H. (2026) https://BioRender.com/lx16hwa.

Interactions between *cis*-regulatory elements and *trans*-acting factors govern splice site selection. *Cis*-elements include canonical splice sites, branch points, polypyrimidine tracts, and regulatory motifs located within exons and introns. These elements establish the structural framework required for spliceosome assembly and splice site recognition. Exonic splicing enhancers (ESEs) and intronic splicing enhancers (ISEs) generally promote spliceosome assembly through recruitment of serine/arginine-rich (SR) proteins, whereas exonic splicing silencers (ESSs) and intronic splicing silencers (ISSs) frequently recruit heterogeneous nuclear ribonucleoproteins (hnRNPs) that repress splice site recognition (Figure 1a) [56].

*Trans*-acting factors, primarily RNA-binding proteins (RBPs), regulate splice site selection through interactions with these *cis*-elements. Among these regulators, SR proteins represent one of the best-characterized families of plant splicing factors. SR proteins generally promote splice site recognition and exon inclusion by facilitating the recruitment of spliceosomal components to nearby splice sites [57]. Structurally, SR proteins typically contain one or two RNA-recognition motifs (RRMs) and a C-terminal arginine/serine-rich (RS) domain involved in RNA binding and protein–protein interactions. Plant genomes contain expanded SR protein families compared with metazoans, suggesting diversification of plant-specific splicing regulation [57]. In addition to AS, SR proteins participate in mRNA export, NMD, mRNA stability, translation, and miRNA biogenesis, thereby linking multiple layers of RNA metabolism [57]. hnRNPs often function antagonistically to SR proteins by repressing splice site usage or promoting exon skipping [56]. Together, the balance between SR proteins and hnRNPs shapes isoform composition across tissues and developmental stages.

Because distinct *trans*-acting factors preferentially recognize specific *cis*-regulatory elements, different combinations of *cis*-elements and RBPs generate highly context-dependent AS patterns and contribute substantially to transcriptome diversity [56,58].

#### 2.3.2. Secondary Structure of RNA in Splice Site Selection

Increasing evidence indicates that RNA secondary structures also contribute to splice site selection and AS regulation. Stem-loop structures, hairpins, and long-range intramolecular RNA interactions can influence splice site accessibility by masking or exposing splice sites and splicing-regulatory motifs [59]. These structural features may additionally affect recruitment of spliceosomal components and RBPs, thereby altering exon inclusion, exon skipping, or intron retention. Such observations support the concept of an “splicing code,” in which both primary nucleotide sequences and higher-order RNA structural features cooperatively determine AS outcomes [56]. Because RNA folding frequently occurs co-transcriptionally, transcription elongation kinetics can further influence RNA structural dynamics and splice site recognition, thereby linking transcriptional regulation to post-transcriptional RNA processing [56,60].

#### 2.3.3. RNA Elongation Rate in Splice Site Selection

In plants, splicing frequently occurs co-transcriptionally, tightly coupling RNA processing with transcriptional dynamics. During transcription, splice sites emerge sequentially from elongating RNA Polymerase II (Pol II), and the transcriptional elongation rate determines the temporal window available for spliceosome assembly and splice site recognition [60,61,62]. Slow transcriptional elongation can provide additional time for recognition of weak splice sites and recruitment of splicing factors, thereby promoting exon inclusion and efficient intron removal. In contrast, rapid elongation may shorten the opportunity for splice site recognition, increasing the likelihood of exon skipping, alternative splice site usage, or intron retention [61,62]. Increasing evidence further suggests that transcriptional kinetics can influence RNA secondary structure formation and the accessibility of *cis*-regulatory elements during co-transcriptional RNA folding. In addition, chromatin organization, nucleosome occupancy, and histone modifications can modulate Pol II elongation dynamics and spliceosome recruitment, further influencing AS outcomes. Together, these observations indicate that co-transcriptional splicing provides an additional regulatory layer integrating chromatin status, transcriptional activity, RNA structural dynamics, and post-transcriptional gene regulation in plants [60,61,62].

#### 2.3.4. Chromatin Organization in Splice Site Selection

Chromatin organization further contributes to splice site selection and spliceosome recruitment. Nucleosome occupancy is frequently enriched over exons relative to introns, suggesting that chromatin architecture contributes to exon definition during transcription [63]. Histone modifications, including H3K36 methylation, have been associated with alternatively spliced regions, further supporting the idea that epigenetic states influence AS outcomes by modulating transcriptional kinetics and the accessibility of splicing regulators [63,64].

Together, these findings demonstrate that pre-mRNA splicing in plants is a highly coordinated regulatory process rather than an isolated RNA-processing event. Splice site selection and AS outcomes are governed through dynamic interactions among *cis*-regulatory elements, RBPs, spliceosomal components, RNA structural remodeling, transcriptional kinetics, and chromatin organization. The integration of these multiple regulatory layers enables highly context-dependent control of transcript isoforms and substantially expands transcriptome and functional diversity in plants (Figure 1b).

### 2.4. Dynamic Regulation of AS

AS is a highly dynamic process regulated by multiple interconnected molecular layers. Splicing patterns vary extensively across tissues, developmental stages, and environmental conditions, reflecting the responsiveness of AS to endogenous and exogenous signals.

Transcriptional dynamics represent a major determinant of splice site selection. RNA Pol II elongation rate influences the temporal availability of splice sites and the recruitment of splicing regulators [60,61,62]. Chromatin organization further modulates splice site accessibility through nucleosome positioning and histone modifications [63]. These co-transcriptional mechanisms allow the integration of transcriptional activity and RNA processing during gene expression regulation.

Post-translational modifications of splicing factors provide additional regulatory flexibility. SR protein-specific kinases and phosphatases dynamically regulate SR protein activity through reversible phosphorylation and dephosphorylation cycles [65,66]. Phosphorylation of SR proteins strongly influences their nuclear localization, RNA-binding affinity, spliceosomal recruitment, and protein–protein interactions [56,65,66]. In addition to phosphorylation, redox-dependent modifications, ubiquitination, and SUMOylation further contribute to the dynamic regulation of spliceosomal proteins and RNA-binding factors [56,67].

Environmental signals frequently trigger large-scale AS remodeling. Temperature fluctuations, drought, salinity, light conditions, and pathogen responses all induce rapid changes in isoform composition. Importantly, many stress-responsive AS events occur independently of transcriptional changes. This demonstrates that AS functions as an autonomous regulatory layer controlling gene-expression plasticity rather than merely representing a secondary consequence of transcriptional regulation.

Recent transcriptomic and proteogenomic studies revealed that AS landscapes are highly tissue-specific and developmentally regulated. Differentially spliced genes (DSGs) often show partial overlap with differentially expressed genes, indicating that AS contributes unique regulatory information beyond transcriptional control [67,68,69,70,71]. Increasing evidence additionally suggests that AS contributes to phenotypic plasticity, environmental adaptation, domestication, and evolutionary divergence among plant populations [2,67].

Together, these observations highlight the dynamic nature of AS and its contribution to transcriptome plasticity across developmental and environmental contexts.

## 3. AS in Plant Development

### 3.1. AS in Early Development and Developmental Initiation

Early developmental stages require precise regulation of gene expression to ensure successful transition from seed dormancy to active growth. AS contributes to these processes by modulating the activity, stability, and regulatory properties of key developmental regulators, particularly those associated with abscisic acid (ABA)-mediated signaling pathways. Through isoform-specific regulation, AS enables plants to fine-tune developmental timing and environmental responsiveness during germination and early seedling establishment [72].

One of the best-characterized examples involves *ABSCISIC ACID INSENSITIVE3* (*ABI3*) in *Arabidopsis*, a master regulator of seed maturation and dormancy [15]. *ABI3* undergoes AS to generate multiple transcript isoforms with distinct transcriptional regulatory capacities. These isoforms differ in the composition of conserved activation domains and DNA-binding-associated regions, thereby altering ABA sensitivity and downstream gene expression during seed maturation and germination [15]. AS-mediated modulation of *ABI3* function is thought to provide flexibility in balancing dormancy maintenance and germination competence under fluctuating environmental conditions. Similarly, ABI5, a basic leucine zipper (bZIP) transcription factor functioning downstream of ABA signaling, also undergoes AS in *Arabidopsis*. Distinct *ABI5* splice variants differentially regulate ABA-responsive gene expression and stress-responsive developmental programs [15].

Isoform-specific changes in transcriptional activity and protein accumulation influence seed germination efficiency and early seedling growth, further illustrating how AS dynamically modulates hormonal signaling during developmental initiation. It should be noted that not all alternatively spliced transcripts necessarily produce functional protein isoforms. In many cases, AS generates transcripts containing premature termination codons that are subsequently degraded through NMD, thereby regulating transcript abundance rather than proteome diversity [3]. Thus, AS-mediated developmental regulation may involve both isoform-specific protein functions and AS-coupled RNA surveillance mechanisms.

Another representative developmental regulator is *DELAY OF GERMINATION1* (*DOG1*), which plays a central role in seed dormancy control in *Arabidopsis* [15]. AS contributes to the generation of multiple *DOG1* transcript isoforms that differ in their relative abundance during seed maturation and imbibition. Functional analyses demonstrated that the balance among *DOG1* isoforms influences dormancy depth and germination timing [15], suggesting that AS functions as an important regulatory mechanism controlling developmental transitions in response to seasonal and environmental cues.

Beyond ABA signaling, AS also modulates cytokinin-mediated developmental regulation. A well-known example is CYTOKININ RESPONSE1 (CRE1), a cytokinin receptor in *Arabidopsis* [16]. AS of *CRE1* generates truncated receptor isoforms through intron-retention events that remove the C-terminal receiver domain required for signal transduction. These truncated proteins retain cytokinin-binding capacity but fail to activate downstream phosphorelay signaling, thereby functioning as dominant-negative decoy receptors [16]. Through this mechanism, AS buffers cytokinin sensitivity and dynamically modulates developmental responsiveness by altering receptor composition and signaling output [16]. The *CRE1* system represents one of the clearest examples of how AS directly reshapes hormone receptor function to fine-tune developmental signaling pathways.

Collectively, these findings demonstrate that AS contributes to early developmental regulation through isoform-specific modulation of hormone signaling pathways, transcription factor activity, and developmental timing mechanisms. Rather than functioning solely as a mechanism for transcript diversification, AS acts as a dynamic regulatory layer that fine-tunes developmental initiation in response to endogenous and environmental signals.

### 3.2. AS in Vegetative Growth and Organ Development

During vegetative growth, plants continuously generate new organs and tissues through coordinated regulation of cell proliferation, differentiation, and developmental patterning. AS contributes extensively to these processes by modulating the expression and functional properties of developmental regulators involved in root architecture, leaf morphogenesis, chloroplast development, and tissue differentiation.

Root development is highly dependent on auxin signaling pathways, and AS regulates multiple components of these pathways. In *Arabidopsis*, *AUXIN RESPONSE FACTOR7* (*ARF7*) and *ARF19*, which are central regulators of lateral root formation, undergo AS to generate isoforms with distinct regulatory properties [17]. Changes in isoform composition can alter transcriptional activity, DNA-binding capacity, and protein–protein interactions with Auxin/INDOLE-3-ACETIC ACID (Aux/IAA) repressors, thereby influencing downstream auxin-responsive gene expression [17]. These isoform-dependent differences affect lateral root initiation and root branching architecture [17], indicating that AS contributes to the spatial and temporal coordination of auxin signaling during root system development.

Although auxin signaling represents a central regulatory pathway controlling lateral root formation, increasing evidence indicates that root developmental plasticity is governed by extensive crosstalk among multiple phytohormone pathways, including cytokinin, abscisic acid, ethylene, and brassinosteroid signaling. Consequently, AS-mediated regulation of auxin-responsive transcription factors is likely integrated with broader hormone signaling networks that collectively determine root architecture and developmental outcomes under changing environmental conditions [73,74]. Further studies are needed to clarify how AS coordinates these interconnected signaling pathways during root development.

AS-mediated regulation of chloroplast development and leaf coloration has also been demonstrated in cultivated crops. In *Lactuca sativa* (lettuce), a CACTA transposon insertion near the *GOLDEN2-LIKE* (*LsGLK*) gene induces aberrant AS, generating defective splice variants associated with pale-green leaf phenotypes [18]. GLK transcription factors are key regulators of chloroplast development, and altered splicing of *LsGLK* drastically reduces the accumulation of functional transcripts without substantially affecting overall transcript abundance. Consequently, chloroplast biogenesis and chlorophyll accumulation are impaired, resulting in altered leaf coloration [18]. This study provides a clear example of how naturally occurring AS variation contributes to developmental diversification and domestication-associated phenotypic variation in crop species.

Transcriptomic analyses suggest that AS may contribute to vegetative organ morphogenesis in woody plants. In *Liriodendron chinense* (Chinese tulip tree), transcriptome-wide analyses identified more than 50,000 AS events associated with leaf development, with intron retention representing the predominant AS type. DSGs were enriched in pathways associated with leaf morphogenesis, developmental regulation, and cellular differentiation [19]. Among these, the transcription factor *AINTEGUMENTA-LIKE5* (*LcAIL5*) undergoes exon-skipping events that generate distinct transcript isoforms exhibiting stage-specific expression patterns during leaf development [19]. The differential accumulation of LcAIL5 isoforms across developmental stages suggests a potential role for AS in the temporal coordination of leaf shape formation and tissue differentiation during morphogenesis.

Together, these studies demonstrate that AS functions as an important regulatory mechanism during vegetative development by modulating developmental regulators, signaling pathways, and tissue-specific gene expression programs. Through isoform-specific regulation, AS contributes to organ formation, morphological diversification, and developmental plasticity across diverse plant species.

### 3.3. AS in Reproductive Development and Floral Transition

The transition from vegetative growth to reproductive development is one of the most extensively studied developmental processes regulated by AS. The regulation of flowering time requires the integration of environmental and endogenous signals, including temperature, photoperiod, circadian rhythms, and hormonal pathways. AS contributes to this integration by modulating the isoform composition and functional properties of central flowering regulators [20].

A classic example is FLOWERING CONTROL LOCUS A (FCA) in *Arabidopsis*, an RBP that regulates flowering time through autoregulatory AS coupled with alternative polyadenylation [21]. *FCA* produces multiple transcript isoforms, but only the full-length isoform encodes a fully functional protein capable of repressing *FLOWERING LOCUS C* (*FLC*), a major floral repressor [21,22]. Premature cleavage and polyadenylation within intronic regions generate truncated nonfunctional *FCA* transcripts, thereby establishing an autoregulatory negative feedback loop that controls *FCA* abundance and flowering timing [22]. This system represents one of the earliest demonstrations of coordinated regulation among AS, 3′ end RNA processing, and developmental timing in plants.

Another well-characterized regulator is FLOWERING LOCUS M (FLM), a MADS-box transcription factor in *Arabidopsis* [3,20]. *FLM* undergoes temperature-dependent AS to generate two major isoforms, *FLM-β* and *FLM-δ*, which exert antagonistic effects on flowering regulation. Under low temperatures, FLM-β accumulates and forms repressive complexes with SHORT VEGETATIVE PHASE (SVP), thereby delaying flowering. Elevated temperatures promote increased accumulation of FLM-δ and additional nonfunctional isoforms that interfere with SVP-mediated repression [3,20]. This temperature-dependent isoform-switching mechanism enables plants to dynamically adjust reproductive timing in response to ambient thermal fluctuations.

Recent studies have further revealed that photoreceptor signaling pathways directly influence AS during the regulation of flowering. In *Arabidopsis*, the blue-light receptor CRYPTOCHROME2 (CRY2) interacts with the splicing factor CRY2 INTERACTING SPLICING FACTOR 1 (CIS1) to regulate temperature-responsive AS of *FLM* [23]. CRY2-mediated recruitment of CIS1 alters splice site selection within *FLM* transcripts under warm temperatures, thereby promoting flowering transition through modulation of *FLM* isoform balance [23]. These findings establish a direct mechanistic connection between light perception, thermosensory signaling, and AS-mediated developmental regulation.

Photoperiod-responsive AS has also been characterized in monocot crops. In rice, the flowering regulator EARLY FLOWERING DATE1 (ELD1) modulates AS of *CIRCADIAN CLOCK ASSOCIATED1* (*OsCCA1)*, a central circadian clock regulator involved in photoperiodic flowering [24]. Different *OsCCA1* splice isoforms exhibit distinct regulatory effects on flowering-associated transcriptional networks, linking circadian regulation with developmental timing in rice [24].

Emerging evidence additionally highlights the importance of splicing factors themselves in reproductive development. In *Arabidopsis*, U2AF65A and U2AF65B, homologs of the conserved U2 auxiliary factor large subunit, regulate flowering time by controlling AS of flowering-associated transcripts upstream of *FLC*-dependent pathways [25,26]. Mutations in these splicing factors alter flowering timing and disrupt normal AS patterns of developmental regulators, demonstrating that core spliceosome-associated proteins directly contribute to developmental decision-making [25,26].

In *Brassica rapa* ssp. *pekinensis* (Chinese cabbage), hydrogen sulfide (H_2_S) signaling promotes flowering by persulfidation of the splicing factor BraATO2 [27]. Persulfidated BraATO2 alters AS patterns of multiple flowering-related genes, including *AGAMOUD-LIKE 31*/*MADS-AFFECTING FLOWERING 2* (*BraAGL31*/*MAF2*) within the *FLC*-like gene family, thereby accelerating flowering transition [27]. This study demonstrates how post-translational modification of splicing factors can directly link environmental signaling pathways to developmental regulation via AS remodeling.

AS also contributes to reproductive organ development by regulating micro-exon splicing. In *Arabidopsis*, the glycine-rich RNA-binding protein 20 (GRP20) regulates micro-exon retention in floral development-associated transcripts [28]. GRP20-dependent AS events affect splice site precision and transcript maturation of genes involved in reproductive development. Loss of *GRP20* function disrupts normal floral organ development and reproductive competence, indicating that precise micro-exon regulation is essential for developmental fidelity during reproductive morphogenesis [28].

Collectively, these studies establish AS as a central regulatory mechanism controlling reproductive transition and floral development. By integrating temperature, photoperiod, circadian signaling, RNA processing, and splicing-factor regulation, AS enables precise coordination of flowering timing and reproductive competence.

### 3.4. AS in Senescence and Late Developmental Processes

Leaf senescence represents the final stage of plant development and involves extensive transcriptional reprogramming associated with nutrient remobilization, stress adaptation, and developmental aging [75]. Increasing evidence indicates that AS contributes substantially to the regulation of senescence-associated pathways through isoform-specific modulation of transcription factors and signaling networks.

A representative example is the *Populus tomentosa* (poplar) NAC transcription factor RESPONSIVE TO DESICCATION 26 (PtRD26), which generates an intron-retaining splice variant (PtRD26^IR^) that lacks part of the DNA-binding domain. PtRD26^IR^ functions as a dominant-negative regulator by forming heterodimers with PtRD26 and other senescence-associated NAC transcription factors, thereby delaying leaf senescence and modulating the expression of senescence-related genes [29]. Similarly, the rice NAC transcription factor ONAC054 produces alternatively spliced isoforms that differ in the presence of a transmembrane domain. These isoforms exhibit distinct subcellular localization and regulatory activities and contribute to ABA-induced leaf senescence, demonstrating how AS-mediated isoform diversification can fine-tune senescence-associated regulatory networks [30].

Hormone-associated AS pathways additionally contribute to late developmental regulation. Jasmonic acid-responsive regulatory networks frequently exhibit extensive AS remodeling during senescence progression, linking stress signaling pathways to developmental aging [76]. These observations support the idea that AS coordinates developmental and environmental regulatory pathways during late stages of plant growth.

AS additionally contributes to organ-level phenotypic plasticity in crop plants. In *Solanum lycopersicum* (tomato), auxin analog treatment induces large-scale transcriptomic and AS remodeling during fruit development [31]. Genome-wide analyses revealed extensive changes in AS patterns associated with carbohydrate metabolism, phenylpropanoid biosynthesis, cell wall remodeling, and ripening-associated pathways. These AS events coincided with substantial changes in fruit morphology, firmness, ripening progression, and metabolic composition, indicating that AS participates in coordinating developmental and metabolic transitions during fruit maturation [31].

Collectively, these findings indicate that AS contributes to senescence progression by regulating transcriptional networks associated with developmental aging, nutrient remobilization, and cellular reprogramming. Through isoform-specific control of regulatory proteins, AS enables flexible modulation of late developmental transitions.

### 3.5. AS as a Regulatory Layer for Developmental Plasticity

Plant development is highly plastic, allowing continuous adjustment of growth and morphology in response to endogenous signals and environmental fluctuations. AS contributes fundamentally to this developmental plasticity by enabling rapid and reversible modulation of gene function at the isoform level.

Across developmental stages, AS dynamically reshapes transcript composition in response to temperature, light conditions, hormonal signaling, and developmental cues. Development-associated AS frequently affects transcription factors, signaling components, chromatin-associated regulators, and RBPs, thereby influencing multiple regulatory layers simultaneously [9]. Importantly, many developmental AS events alter protein domain composition, subcellular localization, RNA-binding activity, or protein–protein interactions, leading to substantial functional diversification without requiring large changes in overall transcript abundance [3,4,13].

Comparative transcriptomic studies revealed that developmental AS patterns are tissue-specific and stage-dependent. These observations support the importance of isoform-level regulation in shaping developmental phenotypes.

**Figure 2:**
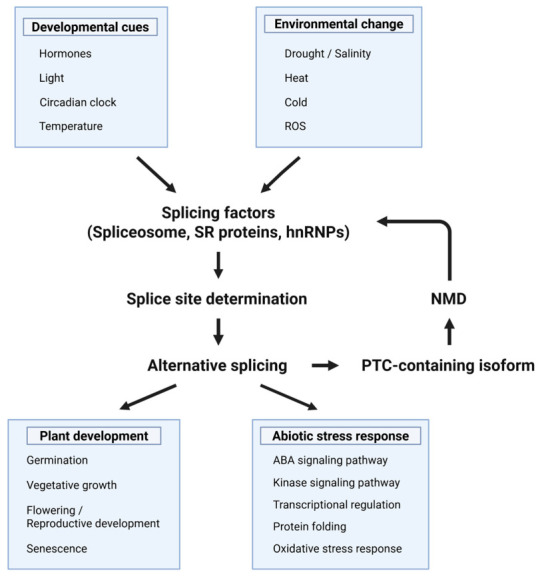
Conceptual model of AS in plant development and abiotic stress responses. AS integrates developmental cues and environmental stress signals to regulate gene expression in plants. External and internal signals influence splicing factors, leading to AS. These isoform-level changes modulate key regulatory pathways involved in plant development and stress responses. Through this mechanism, AS enables dynamic reprogramming of gene expression networks and contributes to plant adaptation under changing environmental conditions. In addition to regulating downstream developmental and stress-responsive pathways, AS may participate in autoregulatory and cross-regulatory feedback circuits through AS-coupled nonsense-mediated decay (AS-NMD). In these circuits, AS-generated premature termination codon (PTC)-containing isoforms are selectively degraded by NMD, thereby modulating the abundance of splicing factors and RBPs. Such feedback mechanisms contribute to splicing homeostasis and the dynamic regulation of AS networks under changing developmental and environmental conditions. Created in BioRender. Seok, H. (2026) https://BioRender.com/sjy79q5.

Collectively, current evidence establishes AS as a major mechanism underlying developmental plasticity in plants. Through isoform diversification and regulatory flexibility, AS enables plants to coordinate developmental progression with environmental responsiveness, thereby enhancing developmental flexibility and adaptive potential (Figure 2, Table 1).

## 4. AS in Abiotic Stress Responses

### 4.1. AS in Osmotic Stress

Drought and salinity are among the most severe abiotic stresses affecting plant growth and productivity. Both conditions impose osmotic stress, ultimately reducing biomass accumulation and yield. To survive under fluctuating environmental conditions, plants rapidly reprogram gene expression networks through multilayered regulatory mechanisms, among which AS has emerged as a major post-transcriptional regulatory process. Numerous transcriptome-wide studies have demonstrated that drought and salinity trigger extensive AS changes in plants, often affecting genes involved in ABA signaling, Mitogen-Activated Protein Kinase (MAPK) pathways, transcriptional regulation, spliceosomal regulation, and epigenetic regulation [33].

#### 4.1.1. Regulation of Abscisic Acid-Mediated Osmotic Stress Response by AS

A central regulatory pathway in osmotic stress responses is ABA signaling. One of the most representative examples of AS in ABA-mediated osmotic stress response is HYPERSENSITIVE TO ABA1 (HAB1) in *Arabidopsis*, a PROTEIN PHOSPHATASE 2C (PP2C) phosphatase that negatively regulates ABA signaling. *HAB1* undergoes AS, generating two major isoforms, *HAB1.1* and *HAB1.2*. *HAB1.1* encodes a full-length functional phosphatase capable of repressing ABA signaling through inhibition of SNF1-RELATED PROTEIN KINASE 2 (SnRK2) activity, whereas *HAB1.2* lacks part of the catalytic domain and exhibits reduced phosphatase activity [34]. The relative abundance of these isoforms changes under stress conditions, suggesting that AS fine-tunes ABA responsiveness by modulating the balance between functional and nonfunctional signaling components [34].

Recent studies have further expanded the mechanistic complexity of these regulatory pathways. In *Arabidopsis*, the PP2C phosphatase HAB2 undergoes AS to generate three splice isoforms, which exhibit distinct biochemical properties and regulatory functions during stomatal regulation and drought adaptation [35]. H_2_S signaling modulates the AS pattern of *HAB2* through the RBP RNA-BINDING MOTIF PROTEIN 25 (RBM25), thereby influencing ABA sensitivity, SnRK2 inhibition, and stomatal closure. HAB2.1 interacts with multiple ABA receptors, including PYL3 and PYL5–13, whereas HAB2.3 loses receptor interaction capacity and exhibits distinct effects on OPEN STOMATA 1 (OST1) phosphorylation and drought tolerance [35]. These findings demonstrate how stress-induced AS can diversify signaling outputs within core ABA regulatory pathways.

#### 4.1.2. Regulation of Mitogen-Activated Protein Kinase-Mediated Osmotic Stress Response by AS

Osmotic stress response is extensively regulated through phosphorylation-mediated activation and inactivation of signaling components, particularly within MAPK signaling pathways [33]. Recent studies indicate that AS-mediated diversification of MAPK-related regulatory components can substantially alter signaling specificity and downstream stress adaptation. In maize, the phosphatase gene *ZmPP2C26* produces two splice isoforms, *ZmPP2C26L* and *ZmPP2C26S*, through atypical AS [36]. The shorter isoform lacks an MAPK-interaction motif and exhibits altered phosphatase activity, substrate specificity, and subcellular localization. ZmPP2C26L localizes to chloroplasts and nuclei and interacts with both ZmMAPK3 and ZmMAPK7, whereas ZmPP2C26S is restricted to the cytosol and nucleus and selectively targets ZmMAPK3. Functional analyses further demonstrated that overexpression of either isoform reduced drought tolerance [37]. Interestingly, ZmPP2C26L and ZmPP2C26S regulate downstream MAPK signaling in an ABA-independent way [36]. This study indicates that AS-mediated diversification of PP2C proteins can substantially alter MAPK-dependent stress signaling pathways.

#### 4.1.3. Regulation of Transcription Factor-Mediated Osmotic Stress Response by AS

Transcription factors play central roles in osmotic stress response by regulating the expression of numerous stress-responsive genes, and increasing evidence indicates that AS substantially contributes to the functional diversification and regulation of these transcriptional regulators under osmotic stress conditions. AS regulates DEHYDRATION-RESPONSIVE ELEMENT-BINDING PROTEIN 2 (DREB2)-type transcription factors involved in drought and heat stress responses. In rice, *OsDREB2B* produces a nonfunctional splice isoform containing a premature termination codon under normal conditions, whereas stress conditions promote accumulation of functional isoforms that activate downstream stress-responsive genes [37]. This isoform-switching mechanism allows rapid activation of stress signaling pathways without requiring extensive transcriptional reprogramming, thereby enabling plants to respond efficiently to sudden environmental changes.

Additional evidence for isoform-specific stress regulation has been reported for MYELOBLASTOSIS ALTERNATIVE SPLICING 1 (ScMYBAS1) in *Saccharum spontaneum* (sugarcane). *ScMYBAS1* generates multiple splice variants with distinct physiological functions. Different splice isoforms affect biomass accumulation and drought tolerance differently, indicating that AS can diversify transcription factor activity and coordinate growth-defense trade-offs under stress conditions [38].

#### 4.1.4. Spliceosomal Regulation of AS in Osmotic Stress Responses

In addition to osmotic stress-responsive signaling and transcriptional regulation, spliceosomal components and splicing factors themselves play central roles in coordinating AS under osmotic stress conditions. SR45, an SR splicing factor in *Arabidopsis*, functions as a key regulator of stress-responsive AS [32]. *SR45* itself undergoes AS to produce two isoforms, *SR45.1* and *SR45.2*, which differ by only a few amino acids but display distinct physiological functions. Functional analyses demonstrated that *SR45.1* efficiently rescues the salt-sensitive phenotype of the *sr45* mutant, whereas *SR45.2* shows limited complementation ability [32]. Similarly, other SR proteins, including SR30, RS40, and RS41, also exhibit stress-responsive AS patterns, and mutations in these genes frequently lead to altered stress tolerance and defects in RNA processing [33]. These findings provide strong evidence that subtle isoform differences generated by AS can substantially alter plant stress tolerance.

Large-scale transcriptomic analyses additionally revealed that AS landscapes vary substantially among crop species and genotypes during osmotic stress adaptation. Genome-wide transcriptomic analyses identified extensive salt stress-induced AS events affecting spliceosome components and SR proteins, including PdRS40, PdRSZ21, PdSR45a, and PdU2AF genes [39]. Interestingly, AS-responsive genes and transcriptionally regulated genes only partially overlapped, suggesting that transcriptional regulation and AS constitute partially independent regulatory layers during salt adaptation [39].

#### 4.1.5. Epigenetic Regulation of AS in Osmotic Stress Response

Epigenetic regulation has also emerged as an important component of stress-responsive AS networks. In *Linum usitatissimum* (linseed), repeated drought stress induced coordinated changes in DNA methylation and AS patterns [77]. Drought-tolerant and drought-sensitive varieties exhibited distinct relationships between differentially methylated genes and DSGs, particularly within stress-responsive pathways [77]. These findings suggest that chromatin status and gene body methylation may influence splice site selection and transcriptome plasticity during repeated drought exposure. Rather than serving merely as a passive consequence of stress, AS functions as an active regulatory layer that enables rapid, flexible adaptation to osmotic stress.

#### 4.1.6. Other Regulatory Mechanisms of Osmotic Stress Response by AS

Recent studies have revealed that stress-responsive AS is regulated not only through core signaling pathways but also through circadian regulation, genotype-specific transcriptome plasticity, and other multilayered regulatory mechanisms.

Drought-responsive AS has been identified in circadian clock-associated genes such as *ZmCCA1* in maize. Stress-induced changes in *ZmCCA1* isoform composition influence drought tolerance and suggest functional interactions between circadian regulation and stress-responsive AS pathways [40]. These observations highlight the integration of environmental signaling, temporal regulation, and post-transcriptional control during stress adaptation.

In rice, comparative long-read transcriptome analyses of salt-tolerant and salt-sensitive cultivars demonstrated extensive genotype-specific AS regulation during early salt stress responses. DSGs were strongly associated with reactive oxygen species (ROS) scavenging, chloroplast regulation, and hormone signaling pathways, suggesting that stress adaptation depends on coordinated transcriptional and post-transcriptional regulation [78].

### 4.2. AS in Heat and Cold Stress

Temperature fluctuations profoundly influence plant growth and survival. Both heat and cold stress disrupt cellular homeostasis, impair protein stability, alter membrane fluidity, and affect metabolic processes. Because environmental temperature can change rapidly, plants require highly dynamic regulatory mechanisms capable of rapidly reprogramming gene expression. AS has emerged as one of the most important mechanisms underlying temperature-responsive transcriptome plasticity [79].

#### 4.2.1. AS in Heat Stress

Heat stress induces widespread AS changes affecting genes involved in transcriptional regulation, RNA processing, protein folding, and stress signaling. Among the best-characterized examples is HEAT SHOCK FACTOR A2 (HSFA2) in *Arabidopsis*, a central transcription factor regulating thermotolerance [41]. Heat stress induces AS of *HSFA2* transcripts, generating isoforms with distinct regulatory properties. Some alternatively spliced isoforms encode truncated proteins lacking transcriptional activation domains, whereas others retain regulatory activity and promote the expression of downstream heat-responsive genes. These isoform-specific differences influence heat stress memory, thermotolerance, and recovery from stress-induced damage [41,42].

Another representative example is bZIP60 in *Arabidopsis*, a transcription factor involved in the unfolded protein response. Unlike conventional spliceosomal AS, *bZIP60* undergoes unconventional cytoplasmic splicing mediated by the endoplasmic reticulum (ER) stress sensor INOSITOL-REQUIRING ENZYME-1 (IRE1) [43,44]. Under heat or ER stress conditions, IRE1 removes a short intron from *bZIP60* mRNA, producing a spliced isoform lacking the transmembrane domain. This structural alteration enables the spliced bZIP60 protein to translocate from the endoplasmic reticulum to the nucleus, where it activates stress-responsive genes involved in protein folding and ER homeostasis [43,44]. The *bZIP60* system represents a striking example of how AS-mediated structural remodeling can alter protein localization and regulatory function during stress adaptation.

Temperature-responsive AS also affects genes associated with photomorphogenesis and circadian regulation. For example, AS of *PHYTOCHROME INTERACTING FACTOR* (*PIF*) genes and *SUPPRESSOR OF PHYA-105 3* (*SPA3)* is influenced by light and temperature conditions, suggesting extensive crosstalk between photoreceptor signaling and stress-responsive RNA processing [45]. In *Arabidopsis*, *PIF3* intron retention within the 5′ untranslated region introduces upstream open reading frames that reduce translation efficiency, thereby linking AS to translational regulation under changing environmental conditions [45].

Temperature-dependent AS is also modulated by kinase signaling pathways. In *Arabidopsis*, the LAMMER kinase AFC2 regulates high-temperature-induced AS by phosphorylating the SR protein RSZ21. AFC2 kinase activity itself is temperature-sensitive, suggesting that AFC2 may function as a thermosensory regulator that links ambient temperature perception to AS regulation [46]. Transcriptome analyses further demonstrated that high temperature induces extensive AS changes in PIF4 target genes, indicating that AS acts as a buffering mechanism that prevents excessive thermomorphogenic responses [47].

Temperature-responsive AS additionally affects protein localization and subcellular dynamics. In *Arabidopsis*, heat stress regulates exon splicing of the DNA glycosylase METHYL-CpG-BINDING DOMAIN PROTEIN 4 LIKE (MBD4L), producing isoforms with distinct subnuclear localization patterns. Exitron retention or removal determines whether MBD4L accumulates in the nucleoplasm or nucleolus, respectively [48]. Heat stress preferentially increases the nucleolar isoform through regulation by the splicing factors NTC-RELATED PROTEIN 1 (NTR1) and RS31 [48]. This study provides one of the clearest examples of how AS can regulate stress adaptation through dynamic control of protein localization. By modulating the isoform composition of transcription factors, signaling components, and RNA-processing factors, AS allows plants to dynamically adjust physiological and developmental programs under fluctuating temperature conditions [48].

Additional evidence indicates that RBPs play important roles in the temporal regulation of thermotolerance. In rice, the glycine-rich RNA-binding proteins OsGRP3 and OsGRP162 regulate heat-responsive AS and contribute to nighttime thermotolerance [49]. The rhythmic expression of *OsGRP3* and *OsGRP162* peaks during nighttime and is further enhanced by heat stress through the evening complex component EARLY FLOWERING 3-2 (OsELF3-2). Double mutants lacking *OsGRP3* and *OsGRP162* exhibit globally altered AS patterns, particularly increased exon skipping, together with severe reductions in heat tolerance and seed-setting rates [49]. These findings demonstrate that circadian regulation and AS are tightly integrated during temperature adaptation.

A recent study further demonstrates direct connections between environmental signaling pathways and AS regulation. In *Arabidopsis*, the RNA helicase U2AF65-ASSOCIATED PROTEIN (UAP56) physically interacts with the light-signaling regulator CONSTITUTIVE PHOTOMORPHOGENIC 1 (COP1) and coordinately regulates large sets of alternatively spliced transcripts during photomorphogenesis [50]. Deep-sequencing analyses revealed substantial overlap between UAP56- and COP1-dependent AS events, suggesting that environmental signal perception is tightly integrated with post-transcriptional gene regulation [50].

Another recent mechanistic study has further revealed that temperature-responsive AS is tightly linked to post-translational regulation of spliceosome components and environmental sensing pathways. In *Arabidopsis*, the PP2A regulatory subunit PP2A B′η interacts with spliceosome-associated proteins, including PRE-mRNA-PROCESSING FACTOR 18a (PRP18a), PRP16, and DEAD-BOX RNA HELICASE 2 (RH2), and mediates their dephosphorylation during heat stress [51]. Loss of *PP2A B′η* results in widespread intron retention in heat-responsive genes and severely compromises thermotolerance, whereas overexpression enhances heat adaptation and efficient intron excision [51]. These findings provide direct evidence that reversible phosphorylation of spliceosome components is essential for heat-responsive AS regulation.

#### 4.2.2. AS in Cold Stress

Cold stress-responsive AS has also been extensively documented in crop species. In *Gossypium hirsutum* (upland cotton), nanopore full-length transcriptome analyses identified more than 21,000 AS events during cotyledon-stage chilling stress [52]. DSGs included ROS-related genes such as *CATALASE* (*CAT*), *ACYL-CoA OXIDASE* (*ACX*), *ALANINE:GLYOXYLATE AMINOTRANSFERASE* (*AGT*), and *SUPEROXIDE DISMUTASE* (*SOD*), as well as genes associated with both C-REPEAT BINDING FACTOR (CBF)-dependent and CBF-independent cold signaling pathways [52]. Interestingly, auxin-related genes such as *IAA16* and *IAA4* were identified as key regulatory hubs that link transcriptional regulation to AS-mediated cold stress responses [52]. These findings suggest that cold-responsive AS may contribute to the balance between growth inhibition and stress adaptation during early seedling development.

In rice, the plant-specific SR protein RS33 regulates stress-responsive AS under low-temperature and salt conditions. Loss-of-function *rs33* mutants exhibit increased sensitivity to chilling stress and salinity, together with widespread alterations in the AS patterns of stress-responsive genes [53]. Interestingly, RS33-dependent AS regulation is more pronounced during low-temperature stress than under salt stress, suggesting that distinct environmental conditions recruit partially specialized AS regulatory networks [53].

GRPs, including GRP7 and GRP8 in *Arabidopsis*, represent another important class of stress-responsive splicing regulators. These proteins participate in cold responses as well as circadian regulation [33,54]. *GRP7* undergoes autoregulatory AS coupled with NMD, forming a negative feedback loop that controls transcript abundance and RNA homeostasis [54]. This mechanism illustrates how AS cooperates with RNA surveillance pathways to maintain dynamic regulation of splicing activity under changing environmental conditions.

### 4.3. AS in Oxidative Stress

Abiotic stresses frequently lead to excessive accumulation of ROS, including superoxide radicals, hydrogen peroxide, and hydroxyl radicals. Although ROS act as important signaling molecules at low concentrations, excessive ROS accumulation causes oxidative damage to proteins, lipids, nucleic acids, and cellular membranes. Plants therefore require tightly coordinated antioxidant systems to maintain redox homeostasis under stress conditions.

AS also affects genes encoding antioxidant enzymes, including *SOD* and *CAT* genes identified in several plant species, including cotton and *Populus trichocarpa* (black cottonwood) [52,55]. Stress-induced changes in isoform composition can alter enzymatic activity, subcellular localization, or protein stability, thereby affecting ROS-scavenging efficiency. Because ROS accumulation influences multiple cellular pathways simultaneously, AS-mediated regulation of antioxidant systems likely contributes to coordinated stress adaptation at the systems level [52,55].

Additional evidence for oxidative stress-responsive AS has emerged from transcriptome-wide studies showing that ROS accumulation broadly affects RNA metabolism and spliceosome function. Oxidative stress can influence the phosphorylation state, localization, and activity of splicing factors, thereby reshaping global AS landscapes [80]. Such observations suggest that ROS signaling and AS regulation are tightly interconnected.

Compared with osmotic and temperature stress responses, relatively few functionally validated examples of oxidative stress-responsive AS have been reported in plants. Consequently, current knowledge is largely derived from transcriptomic and expression-based analyses, highlighting the need for further studies to establish the functional significance of oxidative stress-associated splice variants.

### 4.4. AS in Stress Memory

AS is increasingly implicated in stress memory, a phenomenon in which prior exposure to stress enhances responsiveness to subsequent stress events. Stress memory involves stable or rapidly re-establishable transcriptional and epigenetic states that facilitate adaptive responses during recurring stress exposure. Recent studies indicate that stress-induced AS patterns can persist after stress recovery or be more rapidly reactivated upon repeated exposure [81,82]. ABA-primed barley plants, for example, displayed enhanced differential AS and isoform switching during both drought stress and recovery phases [81]. These transcriptomic changes were associated with improved physiological performance, suggesting that AS contributes to transcriptional memory and adaptive resilience.

At the mechanistic level, stress memory may involve interactions between AS, chromatin remodeling, and RNA surveillance pathways. Stress-induced chromatin modifications can influence transcription elongation and splice site selection, whereas AS-coupled NMD can dynamically regulate transcript abundance during repeated stress cycles [42]. Such multilayered regulation may enable plants to transition rapidly between stress-responsive and recovery states.

### 4.5. Regulation of Splicing Factors Under Abiotic Stress Conditions

Splicing factors themselves are major targets of environmental regulation and function as central regulators of stress-responsive AS networks. Environmental stresses frequently alter the expression, phosphorylation status, subcellular localization, and AS patterns of splicing regulators, thereby reshaping transcriptome-wide splicing landscapes and influencing stress adaptation.

Stress conditions also induce extensive AS changes in components of the spliceosome itself, including snRNP-associated proteins, RNA helicases, and spliceosome assembly factors [3,4,5]. Such regulation may alter spliceosome composition and consequently influence splice site selection across large groups of target genes. Environmental signals further modulate splicing regulation through post-translational modifications such as phosphorylation, ubiquitination, and redox-dependent modifications, which rapidly affect splicing factor activity, stability, RNA-binding affinity, and subcellular localization [57,65,66,67].

Increasing evidence additionally supports extensive crosstalk between AS and transcriptional regulation. RNA Pol II elongation rate, chromatin organization, nucleosome positioning, and histone modifications can all influence splice site recognition and AS outcomes [60,61,62]. Stress-induced chromatin remodeling may therefore indirectly regulate AS by altering transcriptional kinetics and spliceosome recruitment, linking transcriptional and post-transcriptional regulation during stress adaptation [42,63,64].

Collectively, these studies demonstrate that stress-responsive AS is regulated by interconnected mechanisms, including spliceosome remodeling, RBPs, post-translational modifications, chromatin-associated regulation, and environmental signaling pathways. Rather than functioning solely as downstream targets of stress signaling, splicing factors act as dynamic regulatory hubs that integrate environmental and developmental signals to coordinate transcriptome plasticity under stress conditions (Figure 2, Table 2).

## 5. Future Perspectives of AS in Plants

Despite substantial advances in transcriptomic technologies, several methodological challenges continue to complicate the detection and interpretation of AS events. Incomplete genome annotations can hinder the identification of novel splice variants, while batch effects and technical variability may influence AS detection across experiments. Accurate quantification of individual isoforms also remains challenging, particularly for low-abundance transcripts with high sequence similarity. Furthermore, insufficient biological replication can reduce confidence in detected AS events and contribute to inconsistencies among studies. Addressing these limitations will be essential for improving the accuracy, reproducibility, and biological interpretation of future AS analyses.

Despite major progress in transcriptome-wide characterization of AS, several important challenges remain. A major limitation is the difficulty in distinguishing functional splice variants from transcriptional or splicing noise. Although recent proteogenomic studies demonstrated that many alternatively spliced transcripts are translated, the physiological significance of numerous splice isoforms remains incompletely understood [12,83]. Many AS events may exhibit condition-specific, low-abundance, or transient expression patterns that complicate functional validation. Consequently, systematic experimental characterization of isoform-specific functions remains a major priority in the field. Future studies should focus on defining causal relationships between AS regulation and plant phenotypes through rigorous functional validation approaches.

An additional challenge is determining which alternatively spliced transcripts contribute to functional protein diversity. Integrating isoform-resolved transcriptomics with ribosome profiling, quantitative proteomics, and proteogenomics will be essential for distinguishing translated isoforms from transcripts that primarily function through RNA-level regulatory mechanisms, including AS-coupled NMD.

Accurate reconstruction and quantification of full-length transcript isoforms also remain major challenges in AS research. Short-read sequencing technologies often fail to resolve complex transcript structures, particularly for genes exhibiting extensive AS. Long-read sequencing platforms, including PacBio Iso-Seq and Oxford Nanopore technologies, have substantially improved isoform resolution and revealed unexpectedly complex AS landscapes across tissues and conditions [83]. A major priority for future research will be the integration of long-read sequencing with high-depth short-read RNA-Seq datasets. While long-read technologies provide accurate reconstruction of full-length transcript isoforms, short-read sequencing remains advantageous for robust transcript quantification across large sample sets. The combination of these complementary approaches will enable more accurate characterization of AS dynamics across developmental stages and environmental conditions. In parallel, single-cell and spatial transcriptomic approaches are beginning to uncover cell-type-specific and spatially regulated AS programs that were previously inaccessible using bulk transcriptomic analyses [84,85,86].

Technological advances are also transforming functional studies of AS. Emerging genome-engineering approaches, including CRISPR-mediated splice site editing, isoform-specific perturbation, and RNA-targeting technologies, now enable direct manipulation of splicing patterns and experimental investigation of isoform-specific functions [58]. These approaches provide powerful opportunities to dissect the regulatory mechanisms underlying development and stress responses and may ultimately facilitate targeted engineering of agriculturally important traits through manipulation of AS pathways.

In addition to technological advances, future progress in plant AS research will require greater emphasis on experimental validation and cross-study comparability. The development of standardized frameworks for AS event classification, quantification, and reporting would facilitate comparisons among species, tissues, and stress conditions. In addition, the increasing application of single-cell and spatial transcriptomic approaches will facilitate discrimination between genuine stress-induced AS responses and apparent AS changes resulting from shifts in cellular composition. Together, these advances will help establish a more comprehensive and mechanistic understanding of how AS contributes to plant development and environmental adaptation.

Recent advances further suggest that AS contributes substantially to phenotypic plasticity, adaptive evolution, and domestication [2,67]. Population-scale analyses have identified extensive natural variation in AS patterns associated with developmental traits, stress adaptation, and agronomic phenotypes. In rice and other crop species, splice site polymorphisms, structural variants, and splicing quantitative trait loci have been linked to flowering time, metabolism, stress tolerance, and morphological diversification [70,87]. These observations indicate that AS regulation itself represents an important substrate for adaptive evolution and crop improvement.

Beyond fundamental biology, AS represents a promising target for crop improvement. Because AS contributes to stress adaptation, developmental plasticity, metabolic regulation, and environmental responsiveness, targeted manipulation of splicing patterns in key regulatory genes may provide novel strategies for improving stress tolerance, productivity, and phenotypic stability in crops [2,33]. Integrating transcriptomics, proteomics, genetics, computational biology, and genome engineering will therefore be essential for translating mechanistic understanding of AS into practical agricultural applications.

Overall, current evidence supports AS as a central regulatory mechanism operating across multiple layers of plant gene regulation. Continued integration of isoform-resolved transcriptomics, proteogenomics, functional genomics, and genome-editing technologies will be essential for establishing causal links between AS regulation and plant phenotypes and for realizing the full potential of AS in plant biology and crop improvement.

## Figures and Tables

**Table 1:** Alternative splicing (AS) in plant development.

Gene	Species	Developmental Process	AS Feature	Type of Evidence *	Reference
ABI3	*Arabidopsis*	Seed dormancy and germination	Production of multiple transcript isoforms with distinct transcriptional activities	3	[15]
ABI5	*Arabidopsis*	Seed dormancy and germination	Isoform-specific regulation of ABA-responsive transcription	1	[15]
DOG1	*Arabidopsis*	Seed dormancy	Differential accumulation of splice isoforms	3, 4	[15]
CRE1	*Arabidopsis*	Cytokinin signaling during development	Intron retention generating truncated receptor isoforms	3, 4	[16]
ARF7	*Arabidopsis*	Root development	Isoform diversification affecting auxin signaling	1, 2	[17]
ARF19	*Arabidopsis*	Root development	Isoform diversification affecting auxin signaling	1, 2	[17]
LsGLK	Lettuce	Chloroplast and leaf development	Transposon-induced aberrant splicing	3	[18]
LcAIL5	Chinese tulip tree	Leaf morphogenesis	Exon-skipping events during leaf development	1, 2	[19]
FLM	*Arabidopsis*	Thermosensory flowering	Temperature-dependent production of FLM-β and FLM-δ isoforms	2, 3, 4, 5, 6, 9	[20]
SVP	*Arabidopsis*	Thermosensory flowering	Interaction with FLM splice isoforms	1	[20]
FCA	*Arabidopsis*	Flowering regulation	Autoregulatory AS coupled with alternative polyadenylation	2, 3, 4, 5, 6	[21]
FLC	*Arabidopsis*	Flowering regulation	FCA-associated AS regulatory pathway	2, 3, 6	[21,22]
CRY2	*Arabidopsis*	Thermosensory flowering	Regulation of FLM splice site selection	3	[23]
CIS1	*Arabidopsis*	Thermosensory flowering	Regulation of FLM splice site selection	3, 5, 7	[23]
ELD1	Rice	Photoperiodic flowering	Regulation of OsCCA1 AS	3, 5, 7	[24]
OsCCA1	Rice	Photoperiodic flowering	ELD1-dependent AS regulation	2, 3, 4, 6, 7	[24]
U2AF65B	*Arabidopsis*	Flowering regulation	Regulation of flowering-associated AS events	3, 5	[25]
U2AF65A	*Arabidopsis*	Flowering regulation	Regulation of flowering-associated AS events	3, 5	[26]
BraATO2	Chinese cabbage	Flowering regulation	H_2_S-responsive regulation of AS	3	[27]
BraAGL31 /MAF2	Chinese cabbage	Flowering regulation	BraATO2-dependent AS regulation	2, 3, 4, 5	[27]
GRP20	*Arabidopsis*	Reproductive development	Regulation of micro-exon retention	3	[28]
PtRD26	poplar	Leaf senescence	Dominant-negative isoform by intron retention	2, 6, 8	[29]
ONAC054	rice	Leaf senescence	Transmembrane domain-associated AS	2, 6	[30]
ERF-related and ripening-associated genes	Tomato	Fruit development and ripening	Auxin-responsive AS remodeling	1	[31]
SR45	*Arabidopsis*	Vegetative growth and development	Production of SR45.1 and SR45.2 isoforms	2, 3, 4, 5, 6	[32]

* 1, RNA-Seq; 2, isoform quantification; 3, mutant analysis; 4, complementation; 5, overexpression; 6, biochemical assay; 7, protein–protein interaction assay; 8, proteomics; 9, CRISPR splice-site editing.

**Table 2:** AS in plant abiotic stress responses.

Gene	Species	Stress Type	AS Feature	Type of Evidence *	Reference
SR30	*Arabidopsis*	Stress-responsive AS regulation	Stress-responsive AS of SR proteins	1, 2	[33]
RS40	*Arabidopsis*	Stress-responsive AS regulation	Stress-responsive AS of SR proteins	1, 2	[33]
RS41	*Arabidopsis*	Stress-responsive AS regulation	Stress-responsive AS of SR proteins	1, 2	[33]
HAB1	*Arabidopsis*	Drought/Salinity	Generates HAB1.1 and HAB1.2 splice isoforms with distinct phosphatase activities	1, 2, 4	[34]
RBM25	*Arabidopsis*	Drought/Salinity	Modulates HAB2 AS under H_2_S signaling	3, 5	[35]
HAB2	*Arabidopsis*	Drought/Salinity	H_2_S-regulated AS generates HAB2.1–HAB2.3 isoforms	1, 2, 4, 5, 7	[35]
ZmPP2C26	Maize	Drought/Salinity	Generates ZmPP2C26L and ZmPP2C26S isoforms with distinct regulatory properties	2, 4, 5, 7	[36]
OsDREB2B	Rice	Drought/Cold	Stress-induced accumulation of functional splice isoforms	2, 4, 5	[37]
ScMYBAS1	Sugarcane	Drought/Salinity	Multiple splice variants with distinct transcriptional activities	2, 4, 5	[38]
SR45	*Arabidopsis*	Drought/Salinity/Cold	Production of SR45.1 and SR45.2 isoforms with distinct physiological functions	2, 4, 5	[32]
PdRS40	Date palm	Drought/Salinity	Stress-induced AS of splicing factors	1	[39]
PdRSZ21	Date palm	Drought/Salinity	Stress-induced AS of splicing factors	1	[39]
PdSR45a	Date palm	Drought/Salinity	Stress-induced AS of splicing factors	1	[39]
PdU2AF	Date palm	Drought/Salinity	Stress-induced AS of splicing factors	1	[39]
ZmCCA1	Maize	Drought/Salinity	Stress-responsive isoform switching	2, 4, 5	[40]
HSFA2	*Arabidopsis*	Heat Stress	Heat-induced AS generates isoforms with distinct transcriptional activities	2	[41,42]
bZIP60	*Arabidopsis*	Heat/ER Stress	IRE1-mediated unconventional splicing removes transmembrane domain	3, 5	[43,44]
PIF3	*Arabidopsis*	Heat Stress	Temperature-responsive AS affects transcriptional regulation	1	[45]
SPA3	*Arabidopsis*	Heat Stress	Light- and temperature-responsive AS	1	[45]
AFC2	*Arabidopsis*	Heat Stress	Temperature-sensitive kinase regulating AS	3, 5	[46]
RSZ21	*Arabidopsis*	Heat Stress	SR protein phosphorylated by AFC2	3	[46]
PIF4	*Arabidopsis*	Heat Stress	Temperature-responsive AS affects transcriptional regulation	2, 3, 5	[47]
MBD4L	*Arabidopsis*	Heat Stress	Exitron splicing generates proteins with distinct subnuclear localization	2	[48]
NTR1	*Arabidopsis*	Heat Stress	Regulate heat-responsive exitron splicing of MBD4L	3	[48]
RS31	*Arabidopsis*	Heat Stress	Regulate heat-responsive exitron splicing of MBD4L	5	[48]
OsGRP3	Rice	Heat Stress	Regulate diurnal heat-responsive AS	3, 5	[49]
OsGRP162	Rice	Heat Stress	Regulate diurnal heat-responsive AS	3, 5	[49]
UAP56	*Arabidopsis*	Stress-responsive AS regulation	RNA helicase regulating transcriptome-wide AS	3	[50]
PP2A	*Arabidopsis*	Heat Stress	Interacts with spliceosome-associated proteins	3, 5, 6	[51]
CAT	Cotton	Cold Stress	Extensive cold-induced differential AS	1	[52]
ACX	Cotton	Cold Stress	Extensive cold-induced differential AS	1	[52]
AGT	Cotton	Cold Stress	Extensive cold-induced differential AS	1	[52]
SOD	Cotton	Cold Stress	Extensive cold-induced differential AS	1	[52]
IAA4	Cotton	Cold Stress	AS-associated auxin signaling regulation	1	[52]
IAA16	Cotton	Cold Stress	AS-associated auxin signaling regulation	1	[52]
APX	Cotton	Oxidative Stress	Stress-dependent AS alters transcript composition	3	[52]
RS33	Rice	Stress-responsive AS regulation	Regulates AS of stress-responsive genes under salt and cold stress	2, 3, 5, 6	[53]
GRP7	*Arabidopsis*	Stress-responsive AS regulation	Autoregulatory AS coupled with NMD	2, 3, 5, 6	[54]
GRP8	*Arabidopsis*	Stress-responsive AS regulation	Autoregulatory AS coupled with NMD	1	[54]
CAT	Black cottonwood	Oxidative Stress	Stress-dependent AS alters transcript composition	1, 2	[55]

* 1, RNA-Seq; 2, isoform quantification; 3, mutant analysis; 4, complementation; 5, overexpression; 6, biochemical assay; 7, protein-protein interaction assay.

## Data Availability

No new data were created or analyzed in this study. Data sharing is not applicable to this article.
